# Fisetin exerts antihyperalgesic effect in a mouse model of neuropathic pain: engagement of spinal serotonergic system

**DOI:** 10.1038/srep09043

**Published:** 2015-03-12

**Authors:** Xin Zhao, Chuang Wang, Wu-Geng Cui, Qing Ma, Wen-Hua Zhou

**Affiliations:** 1 Department of Pharmacology, Ningbo University, School of Medical Science, Ningbo, Zhejiang province 315211, China; 2 Zhejiang Province Key Laboratory of Pathophysiology, Ningbo University, School of Medical Science, Ningbo, Zhejiang province 315211, China

## Abstract

Fisetin, a natural flavonoid, has been shown in our previous studies to exert antidepressant-like effect. As antidepressant drugs are clinically used to treat chronic neuropathic pain, this work aimed to investigate the potential antinociceptive efficacies of fisetin against neuropathic pain and explore mechanism(s). We subjected mice to chronic constriction injury (CCI) by loosely ligating the sciatic nerves, and Hargreaves test or von Frey test was used to assess thermal hyperalgesia or mechanical allodynia, respectively. Chronic fisetin treatment (5, 15 or 45 mg/kg, p.o.) ameliorated thermal hyperalgesia (but not mechanical allodynia) in CCI mice, concomitant with escalated levels of spinal monoamines and suppressed monoamine oxidase (MAO)-A activity. The antihyperalgesic action of fisetin was abolished by chemical depletion of spinal serotonin (5-HT) but potentiated by co-treatment with 5-HTP, a precursor of 5-HT. Moreover, intraperitoneal (i.p.) or intrathecal (i.t.) co-treatment with 5-HT_7_ receptor antagonist SB-258719 completely abrogated fisetin's antihyperalgesia. These findings confirm that chronic fisetin treatment exerts antinociceptive effect on thermal hyperalgesia in neuropathic mice, with spinal serotonergic system (coupled with 5-HT_7_) being critically involved. Of special benefit, fisetin attenuated co-morbidly behavioral symptoms of depression and anxiety (evaluated in forced swim test, novelty suppressed feeding test and light-dark test) evoked by neuropathic pain.

Neuropathic pain is usually an enduring and devastating condition resulting from damage to nociceptive pathways in the peripheral or central nervous system^1^. This disease represents a significant challenge in clinical practice due to, at least partly, its multi-facet etiologies, symptoms and underlying mechanisms^2^. Different from acute pain management that relies on conventional analgesics such as opioids and non-steroidal anti-inflammatory drugs^3^,^4^, the most effective pharmacological treatment of neuropathic pain are based on drugs initially developed to treat other CNS diseases, i.e. antidepressants and anticonvulsants^5^. Despite being ranked as first-line drugs, these agents (antidepressants and anticonvulsants) could not fully satisfy the clinical need of quenching pain in neuropathic patients, because of modest efficacy, extensive limitations, undesirable side effects and poor patient compliance^5^,^6^, thus posing the necessity to develop novel analgesics including herbal and complementary medicine.

Fisetin (3,3′,4′,7-tetrahydroxyflavone, for the chemical structure to see Fig. 1A) is a flavonoid rich in strawberries and other edible fruits or vegetables^7^. It has a wide variety of pharmacological activities such as anti-allergic^8^, cancer chemo-preventive^9^ and neuroprotective activities^10^. Recently, we originally revealed that fisetin possesses anti-depression property in experimental animals^11^. As antidepressant drugs are clinically used to treat persistent and neuropathic pain, we reasoned that fisetin may also have therapeutic potentials in fighting chronic neuropathic pain. Therefore, the first aim of the present study is to investigate the possible antinociceptive capacity of fisetin in a mouse neuropathic pain model produced by loosely ligating the sciatic nerves (CCI model). Furthermore, we explored the mechanisms underlying the actions of fisetin in the context of neuropathic pain. We hypothesized involvement of monoaminergic system, since it is not only substantially implicated in the descending pain regulation^12^, but is responsible for the antidepressant-like action of fisetin^11^. Finally, based on the well known co-morbidity between neuropathic pain and mood disorders, esp. depression and anxiety^13^,^14^,^15^, we also evaluated whether fisetin engenders beneficial effects on the co-morbidly behavioral phenotypes of depression and anxiety evoked by chronic neuropathic pain.

## Methods

### Animals

Adult male C57BL/6J mice (6–7 weeks old upon arrival and obtained from the Laboratory Animal Center of Chinese Academy of Sciences) were used in this study. Animals were group-housed (4–6 per cage) with food and water available *ad libitum*, and kept in controlled laboratory conditions with the temperature maintained at 22 ± 0.5°C and a relative humidity of 60 ± 2% in 12 h light cycles (lights on at 07:00 AM). Experimental behavioral tests were performed in a soundproof and air-regulated room and were done in blind respect to surgical procedure and drug treatment. All experiments and animal handing were in accordance with the European Communities Council Directive of 24 November 1986 (86/609/EEC) and approved by the Ningbo University Animal Committee. The authors tried all efforts to minimize the number of animals used and their suffering.

### Chronic constriction injury (CCI) procedure

The chronic constriction injury (CCI) model was used to induce neuropathic pain as delineated by Bennett and Xie^16^ for rats with minor modifications by us for mice^17^. Briefly, mice were anesthetized by intraperitoneal (i.p.) injection of pentobarbital sodium (65 mg/kg) and the right common sciatic nerve was exposed at the level of the mid thigh of hindpaw. Proximal to the sciatic nerve trifurcation, three ligations (with 1 mm spacing) were loosely tied until a brief twitch was seen in the right hindlimb. The surgical area was dusted with streptomycin and the incision was sutured. In sham-operated animals, the sciatic nerve was isolated but not ligated. All surgical procedures were performed by the same researcher. Animals that did not develop an enhanced response to thermal stimuli (hyperalgesia) following CCI-lesion or showed self mutilation were excluded from the present study. Lack of behavioral hypersensitivity is defined as a decrease of thermal latency less than 20% of the average value of the sham-operated group. Dropouts were not replaced and the rate of exclusion in our laboratory is generally no more than 5%.

### Pharmacological treatments

**Figure 1:**
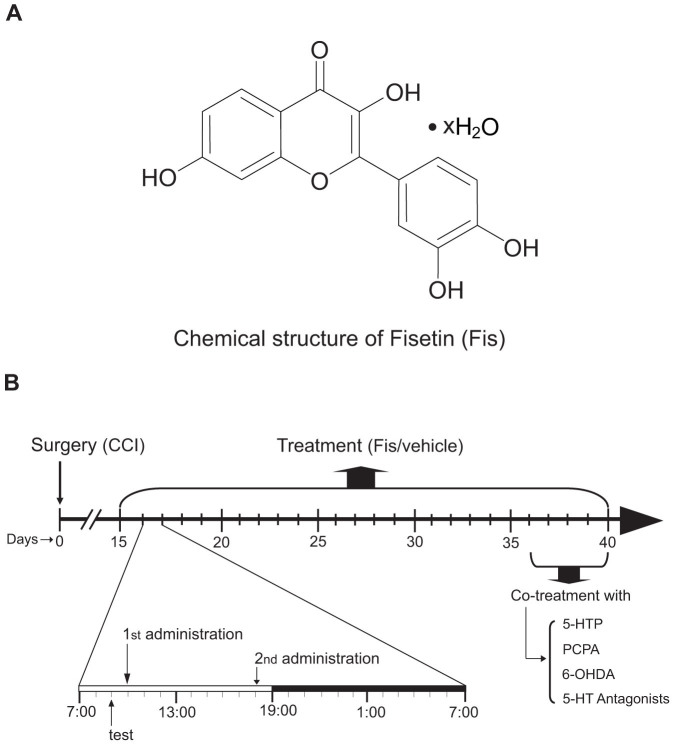
Chemical structure of fisetin (Fis) and schematic of fisetin administration. (A) Chemical structure of fisetin. (B) Fifteen days following the chronic construction injury (CCI), we started chronic treatment with fisetin (5, 15 and 45 mg/kg, p.o., twice a day, at 10:00 and 18:00 respectively). During chronic fisetin treatment, behavioral testes were done just 1 h before the morning drug administration. After 3 weeks of fisetin treatment, 5-HTP, PCPA, 6-OHDA or 5-HT antagonists were co-administered with fisetin.

The treatment with fisetin (p.o., via gavage, with a volume of 10 ml/kg) began 15 days after the surgical procedure (CCI or sham-operation), when the CCI mice exhibited obvious nociceptive hypersensitivity i.e. thermal (heat) hyperalgesia and mechanical allodynia. For chronic treatment, the sham and CCI mice received two oral administrations (morning and evening, as shown in Fig. 1B) of fisetin or vehicle per day for 21 consecutive days (i.e. from day-15 to day-35 after CCI surgery). After 3 weeks of fisetin or vehicle treatment, the mice were co-administered with 5-hydroxytryptophan (5-HTP, a precursor of 5-HT) or one of the 5-HT receptor antagonists (Fig. 1B). These antagonists were: WAY-100635 (5-HT_1A_ receptor antagonist), ritanserin (5-HT_2A/2C_ receptor antagonist), ondansetron (5-HT_3_ receptor antagonist) and SB-258719 (5-HT_7_ receptor antagonist). The doses of these drugs were selected on the basis of our previous studies (for WAY-100635, ritanserin and ondansetron, see Zhao et al.^17^) and other reports (for 5-HTP, see Liang et al.^18^; for SB-258719 see Brenchat et al.^19^) with minor revisions to ensure a paucity of intrinsic activity for these antagonists per se in the present behavioral tests. For i.p. injection, these agents were administered in a volume of 10 ml/kg. For repeated co-administration with fisetin, these agents were injected twice per day, 30 min before fisetin treatment^20^. SB-258719 was purchased from Tocris Bioscience and the other drugs were from Sigma-Aldrich. All the drugs were dissolved or diluted in sterile saline with the exception of fisetin, whose vehicle was saline containing 0.5% sodium carboxymethyl cellulose.

### Intrathecal, intracerebroventricular and intraplantar injection

To localize the monoamine receptors possibly involved, intrathecal (i.t.), intracerebroventricular (i.c.v.) or intraplantar (i.pl.) injection of 5-HT_7_ receptor antagonist SB-258719 was performed following 3 weeks of fisetin treatment. We did two i.t., i.c.v. or i.pl. injections on day-36, 30 minutes before the morning and the evening treatments with fisetin, respectively. The mice were then tested for pain-related behaviors on the following morning, i.e. 39 h following the last administration of fisetin alone. These procedures were based upon previous studies on the analgesic mechanisms of antidepressants^20^,^21^.

Intrathecal injections were done as described by Hylden and Wilcox^22^. Briefly, a 27-gauge needle connected to a 50 μl Hamilton syringe was inserted into the sub-arachnoidal space, between the L5 and L6 vertebrae. The placement of the needle was verified via the induction of a tail flick movement. A volume of 10 μl was used for i.t. injection.

The procedures for intracerebroventricular injections were based on the methods described by Haley and McCormick^23^ and modified by our previous study^17^. In short, during ether anesthesia, mice were grasped firmly by the loose skin behind the head. A 29-gauge needle connected to a 50 μl Hamilton syringe was inserted perpendicularly through the skull and no more than 2 mm into the brain of the mouse. The injection site was 1 mm lateral to the midpoint on a line drawn to the anterior base of the ears. Injections were performed into the lateral ventricle. After behavioral tests, the mice were decapitated rapidly and the brains were removed and frozen in −70°C. The brains were sectioned (10 μm thickness) in the coronal plane and the sections were stained by cresyl-violet to verify the injection site. The injection success rate in our laboratory was generally more than 95%.

In a separate set of experiments, intraplantar injections were performed under ether anesthesia. A 29-gauge injection needle punctured the ipsilateral plantar skin and was placed in the subcutaneous space just proximal to the footpads. A volume of 10 μl was used for i.pl. injection.

### Chemical depletion of descending noradrenaline (NA) and serotonin (5-HT)

After treatment with fisetin or vehicle for 3 weeks, pharmaceutical manipulations were performed in mice to deplete spinal NA or 5-HT. For chemical denervation of spinal noradrenergic transmission, the mice were injected intrathecally with catecholaminergic neurotoxin 6-OHDA (20 μg per mouse), which was dissolved in 5 μl of 0.9% saline containing ascorbic acid (100 μg/ml)^17^,^24^. To ablate descending 5-HT, *p*-chlorophenylalanine (PCPA, an inhibitor of serotonin synthesis), suspended in 0.5% gum acacia/physiological saline, was administered i.p. for five consecutive days at a dose of 300 mg/kg/day^25^.

### Behavioral Tests

Thermal hyperalgesia and mechanical allodynia were used as outcome measures of pain-related behaviors in mice and were evaluated by Hargreaves test and von Frey test, respectively. The depression-like behaviors in mice were measured by forced swim test (FST) and novelty suppressed feeding test (NSFT), while the anxiety-like behaviors were assayed by light-dark test (LDT) and NSFT. In FST, NSFT and LDT, the animals were tested only once, i.e. the different time points of tests were done on independent sets of animals. In Hargreaves test and von Frey test, the animals were tested consecutively in a time-course fashion. To investigate the acute actions of fisetin, the behavior tests were performed at different time points (e.g. 0.5, 1, 2, 3 and 4 h) after a single fisetin administration on day 15. In the experiment settings for chronic fisetin treatment, the behavioral tests were done 1 hour before the morning fisetin treatment (1^st^ fisetin treatment on the day, shown in Fig. 1B) as reported by previous studies^20^,^26^.

Hargreaves test: Thermal (heat) hyperalgesia was assessed based on the Hargreaves procedure^27^, using a plantar test apparatus (Ugo Basile, Italy) to determine the hindpaw withdraw latency to a thermal stimulus (radiant heat). Mice were placed in small Plexiglas cubicles and allowed to acclimatize for at least 20 min before testing. A radiant heat source with constant intensity (approximately 10 s on average in naïve mice) was focused on the hindpaw of mice and the thermal latency (withdraw latency) was defined as the time (in seconds) from initial heat exposure to the withdrawal of the hind paw. The thermal latency was determined in triplicate for each animal, with 5-min intervals to prevent thermal sensitization and behavioral disturbances. A cut-off time of 22 s was set to prevent tissue damage in the absence of response.

Von Frey test: Mechanical allodynia was tested using a series of von Frey monofilaments (Stoelting USA) and results (mechanical threshold) were expressed as grams. Mice were placed in transparent Perspex cubicles with an elevated wire mesh bottom, and allowed to habituate for at least 20 min before testing. The filaments were applied perpendicularly to the plantar surface of hind paw in a series of ascending forces (equivalent to 0.16, 0.4, 0.6, 1, 1.4, 2, 4, 6, 8, 10 g force, respectively). Each filament was tested five times per paw, and the mechanical threshold was defined as the minimal force that caused at least three withdraws observed out of five consecutive trials^17^.

Forced swim test (FST): FST was conducted according to the method established by Porsolt et al^28^. Briefly, mice were individually plunged into vertical Plexiglas cylinders (height: 25 cm and diameter: 10 cm), which were filled with water (depth: 10 cm and temperature: 24 ± 1°C), for 6 min. Mice were deemed immobile when they floated passively, without motion except for making only necessary movements to keep their heads above the water. The immobility time was recorded during the last 4 min of the total 6 min trial.

Novelty suppressed feeding test (NSFT): NSFT reveals both depression and anxiety-like properties in rodents. The testing apparatus consists of a 40 × 40 × 30 cm plastic box with floor covered with 1 cm sawdust. Food was removed from the home cage 24 h before the test. At the beginning of testing, a pellet of food was placed on a paper in the center of the box. A mouse was then placed in a corner of the box and the latency to first contact or eat the pellet was recorded within a 5-min period. This procedure elicits a conflict between the drive to eat the food and the fear of venturing in the center of the box^29^.

Light-dark test (LDT): The apparatus consists of light and dark boxes (25 × 18 × 20 cm each) connected by a dark tunnel (9 × 7 × 6 cm). The lit compartment was brightly illuminated (1500 lux). Mice were placed in the dark compartment in the beginning of the test and the time spent in the lit compartment was recorded during 5 min.

Locomotor activity test: The assessment of locomotor activity was performed in mice according to our previous studies^11^. Locomotor activity was measured with an ambulometer with five activity chambers (JZZ98, Institute of Materia Medica, Chinese Academy of Medical Sciences, China). Mice were placed in the center of the chambers, with their paws connected or disconnected with the active bars producing random configurations that were converted into pulses. The pulses, which are proportional to the locomotor activity of the mice, were automatically recorded for the cumulative total counts of motor activity. Mice were placed in the test chamber 10 min prior to the recording session and then locomotion counts were recorded for 5 min.

### Measurement of monoamines (metabolites) and monoamine oxidase (MAO) activity

Following 3 weeks fisetin or vehicle treatment, the mice were decapitated and the lumbar enlargements (approximately 1 cm) of their spinal cords were rapidly dissected out and collected. The contents of monoamines (NA, serotonin and dopamine) and their metabolites (5-HIAA and DOPAC) were measured using high-performance liquid chromatography (HPLC) with electrochemical detection (for details see Zhao et al.^17^). The MAO activity was assessed according to our previously established protocol (for details see Zhen et al.^11^).

### Statistical Analysis

All values are presented as the mean ± S.E.M. Data were analyzed by multifactor analysis of variance (ANOVA) or one-way ANOVA. For multifactor-ANOVA, for example, the surgery (sham or CCI) and the treatment (vehicle or drug administration) were done as between-group factors. When needed, the time of measurement (time-course data) was done as a within-subject factor. The Duncan test was used for post hoc comparisons. For one-way ANOVA, Student-Newman-Keuls test was used for multiple comparisons, followed by Student's test to evaluate the difference between two groups at the same time. Differences with *p* < 0.05 were considered statistically significant.

## Results

### Chronic fisetin treatment ameliorated neuropathic hyperalgesia to thermal (heat) stimuli in mice with mononeuropathy

**Figure 2:**
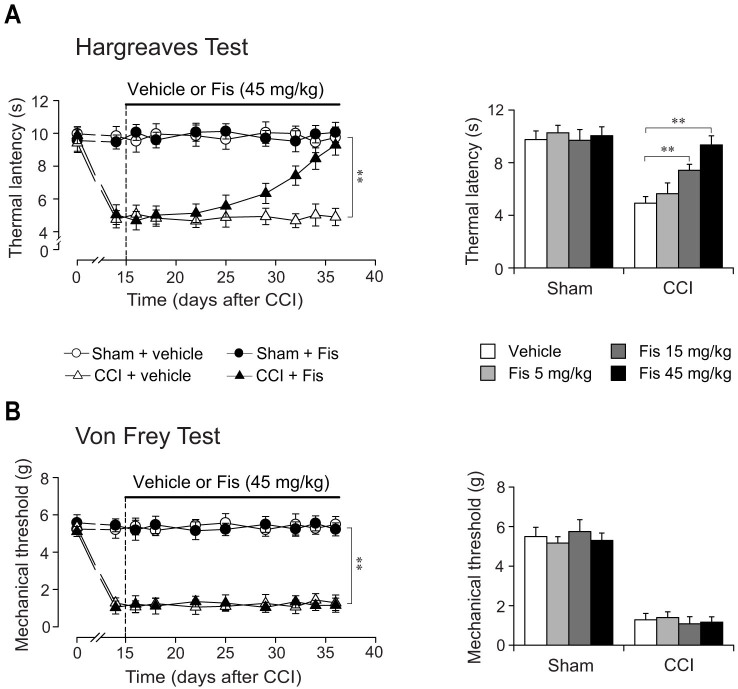
Effects of chronic fisetin (Fis) treatment on mechanical allodynia and thermal hyperalgesia in neuropathic (CCI) mice. The fisetin treatment started 15 days following CCI or sham surgery. (A) Chronic fisetin treatment (45 mg/kg, p.o., twice per day for 21 days) corrected thermal hyperalgesia in neuropathic mice (left panel for time-course) and this antihyperalgesia was dose-dependent (5, 15 and 45 mg/kg, right panel corresponding to values determined on day 36), without affecting the measures in sham-operated mice. (B) Chronic fisetin treatment (5, 15 and 45 mg/kg, p.o.) did not affect the mechanical sensitivity in von Frey test regardless of sham-operated or CCI-injured mice. (A) and (B) Left panel: open circles represent sham + vehicle group; solid circles represent sham + Fis group; open triangles represent CCI + vehicle group; solid triangles represent CCI + Fis group. Data are expressed as mean ± SEM (n = 9–12 per group), evaluated by multifactor ANOVA followed by Duncan test and one-way ANOVA followed by Student-Newman-Keuls test.

The neuropathy induced by peripheral nerve injury (CCI) produced in mice ipsolateral (but not contralateral paw, data not shown) thermal hyperalgesia and mechanical allodynia, which were persistent and maintained throughout the experiment (Fig. 2A and B). In contrast, the nocifensive indices to mechanical and thermal stimuli were not altered by sham-surgery operation.

**Figure 3:**
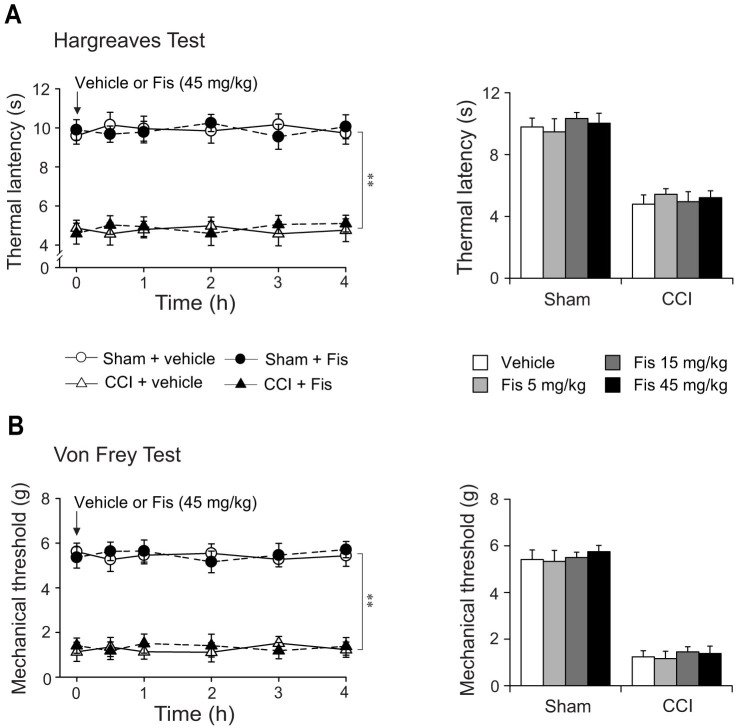
Effect of acute fisetin (Fis) administration on thermal latency and mechanical threshold in sham and CCI mice. Acute fisetin administration started 15 days after CCI surgery. Following baseline (0 h) assay of thermal (A, Hargreaves test) and mechanical (B, von Frey test) sensitivity, mice (sham or CCI) were administered with fisetin (45 mg/kg, p.o.) and then tested at 0.5, 1, 2, 3 and 4 hour after fisetin administration. (A) Acute fisetin treatment did not impact on the thermal sensitivity in both sham and CCI mice. (B) There is no alteration in mechanical sensitivity after single treatment with fisetin. (A) and (B) Left panel: open circles represent sham + vehicle group; solid circles represent sham + Fis group; open triangles represent CCI + vehicle group; solid triangles represent CCI + Fis group. Data are expressed as mean ± SEM (n = 9–12 per group), assessed by multifactor ANOVA followed by Duncan test.

After 3 weeks treatment, fisetin markedly attenuated in CCI mice the pronounced thermal hyperalgesia (F_7,284_ = 12.6, *p* < 0.01; Fig. 2A) in a dose-dependent manner, with significant difference at doses of 15 mg/kg and 45 mg/kg (increase in thermal latency by 52.9% and 90.2%, respectively). At the highest dose of 45 mg/kg, fisetin showed recuperating effects on the thermal hyperalgesia in CCI mice, without influencing the measures in sham-operated mice (Fig. 2A). Nevertheless, the same fisetin regimen did not impact on the nociceptive sensitivity to mechanical stimuli in both sham and CCI mice (Fig. 2B). We also evaluated the acute effects of fisetin on nociceptive behaviors in sham and CCI mice. As shown in Fig. 3, acute fisetin treatment (5, 15 and 45 mg/kg, p.o.) did not influence the nociceptive sensitivity in Hargreaves test and von Frey test regardless of sham or CCI mice.

### Chronic fisetin treatment increased the levels of spinal monoamines and decreased monoamine oxidase (MAO) activity in neuropathic mice

As the monoaminergic system, a key component of descending inhibition in pain modulation^12^, is implicated in the antidepressant-like action of fisetin^11^, we reasoned that the modulation of these descending aminergic pathways by fisetin may be a mechanism underlying its antihyperalgesia. Therefore, we investigated the effects of repeated fisetin treatment on the levels of monoamines and their metabolites in mouse spinal cord. As shown in Table 1, chronic treatment with fisetin (5, 15 and 45 mg/kg) dose-dependently increased, in CCI mice, the contents of serotonin in the spinal cord (by 7%, 27.7% and 55.2%, respectively) and concurrently decreased the ratio of 5-HIAA/5-HT (by 3.5%, 23.5% and 40%, respectively) compared with vehicle treatment. However, fisetin at the highest dose of 45 mg/kg did not alter in sham mice the levels of spinal serotonin and the ratio of 5-HIAA/5-HT. In CCI mice, there was a trend towards escalated NA levels in spinal cord following fisetin treatment, with a marginal difference at the highest dose of 45 mg/kg (increased by 11.2% relative to vehicle). Additionally, we observed a sharp serotonin-fall in vehicle-treated neuropathic mice (compared with vehicle-treated sham mice), which is congruent with the previous study by Vogel et al.^30^ and implies that the insufficiency of serotonin in spinal cord may correlate with the neuropathy induced by peripheral nerve injury, or this aminergic drop is the sequelae following CCI-lesion.

Potentiation of monoamine neurotransmitters can be obtained either by inhibiting their reuptake or by reducing their metabolism via monoamine oxidase (MAO) pathway. The escalated spinal 5-HT contents and depressed ratio of 5-HIAA/5-HT in CCI mice, following fisetin treatment, indicate the alteration of monoamine metabolism may be more likely responsible for the antinociceptive actions of fisetin. The monoamine in CNS is metabolized by two isoforms of MAO (MAO-A and MAO-B), with MAO-A preferentially metabolizing serotonin and NA while MAO-B dealing with NA and dopamine. We then evaluated the effect of chronic fisetin treatment on the MAO-A and MAO-B activities in mouse spinal cord. As shown in Table 2, the activity of MAO-A but not MAO-B increased markedly when the mice were rendered neuropathic by CCI-lesion, which is in congruent with a recent study by Villarinho et al.^31^. Chronic treatment of CCI mice with fisetin (5, 15 and 45 mg/kg) dose-dependently inhibited the escalated spinal MAO-A activity, and the MAO-A activity is reduced to levels observed in non-injured animals following the treatment with fisetin at the dose of 45 mg/kg. In contrast, fisetin treatment did not impact on MAO-B activity in both sham and CCI mice.

### The antihyperalgesic effect of fisetin was abrogated by chemical depletion of spinal 5-HT, but potentiated by co-treatment with 5-HTP

**Figure 4:**
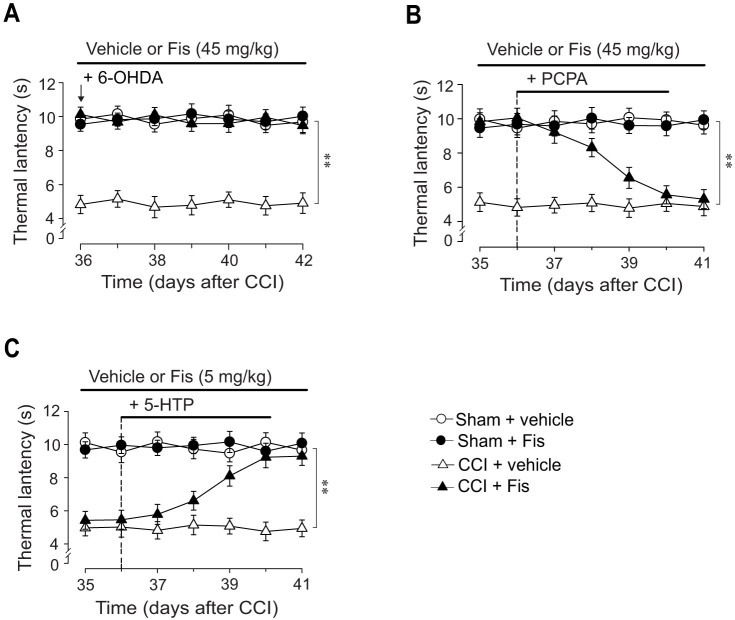
Effects of co-administration of PCPA, 6-OHDA and 5-HTP on the antihyperalgesic effect of fisetin (Fis). To deplete spinal NA or 5-HT, 6-OHDA (20 μg per mouse, i.t.) or PCPA (300 mg/kg, i.p.) was injected once or for 5 consecutive days, respectively. (A) Depletion of NA by 6-OHDA did not influence the measures in Hargreaves test in both sham and CCI mice. (B) Depletion of 5-HT by PCPA abolished the antinociceptive action of fisetin on thermal hyperalgesia in CCI mice, without influencing the thermal sensitivity in sham and vehicle-treated CCI mice. (C) The antihyperalgesic effect of fisetin was potentiated by co-treatment with 5-HTP. Administration of 5-HTP (10 mg/kg, i.p.) or fisetin at ineffective dose (5 mg/kg, p.o.) alone did not exhibit any antihyperalgesic effect in the Hargreveas test regardless of sham or CCI mice, but co-administration of them engendered remarkable antihyperalgesic effect in CCI (not sham) mice. Open circles represent sham + vehicle group; solid circles represent sham + Fis group; open triangles represent CCI + vehicle group; solid triangles represent CCI + Fis group. Data are expressed as mean ± SEM (n = 9–12 per group), assessed by multifactor ANOVA followed by Duncan test.

To determine whether the monoamine system is causally implicated in the antihyperalgesia by fisetin, we investigated the effects of depleting spinal 5-HT or NA on the antihyperalgesic effect of fisetin. It has been reported that a single intrathecal (i.t.) injection of 6-OHDA or consecutive intraperitoneal (i.p.) injections of PCPA reduced spinal NA or 5-HT content, respectively^24^,^25^. Consistently, we also observed a significant reduction of spinal NA or 5-HT content^17^, after a single 6-OHDA (20 μg per mouse) or consecutive PCPA (300 mg/kg, i.p., for 5 consecutive days) administrations, respectively. We thus evaluated the effect of depletion of descending NA and 5-HT, by 6-OHDA and PCPA respectively, on the antinociceptive effects of fisetin. As shown in Fig. 4A, 6 days after a single administration, 6-OHDA did not affect the antihyperalgesic effect of fisetin. Nevertheless, this antihyperalgesia was totally lost when spinal 5-HT was depleted by consecutive PCPA co-administration (F_6,231_ = 10.4, *p* < 0.01; Fig. 4B). In light of these results, it could be presumed that serotonergic mechanism is specifically and causally responsible for the antihyperalgesic action of fisetin. Of note, chemical depletion of spinal 5-HT and NA by PCPA and 6-OHDA, respectively, did not change the measures of pain-related behaviors in sham and vehicle-treated CCI mice (Fig. 4A and B), which is congruent with other reports^24^,^25^ and indicative of lacking intrinsic effect for PCPA and 6-OHDA at doses used here in both sham and neuropathic mice.

To further support the crucial role for serotonergic system in the antihyperalgesic action of fisetin. We examined whether fisetin antihyperalgesia can be influenced by co-treatment with 5-HTP, a precursor of serotonin. As shown in Fig. 4C, a single administration of 5-HTP (10 mg/kg, i.p.) or ineffective dose of fisetin (5 mg/kg, p.o.) did not influence the parameters measured in the Hargreaves test for both sham and CCI mice. However, co-administration of them yielded obvious antihyperalgesic effect in CCI mice (Hargreaves test: F_6,228_ = 9.1, *p* < 0.01), pointing to a potentiating effect when fisetin was co-treated with 5-HTP. These results validate the serotonergic mechanism underlying the antihyperalgesic action of fisetin.

### Spinal 5-HT_7_ receptors were responsible for the antinociceptive effects of fisetin on thermal hyperalgesia in neuropathic mice

**Figure 5:**
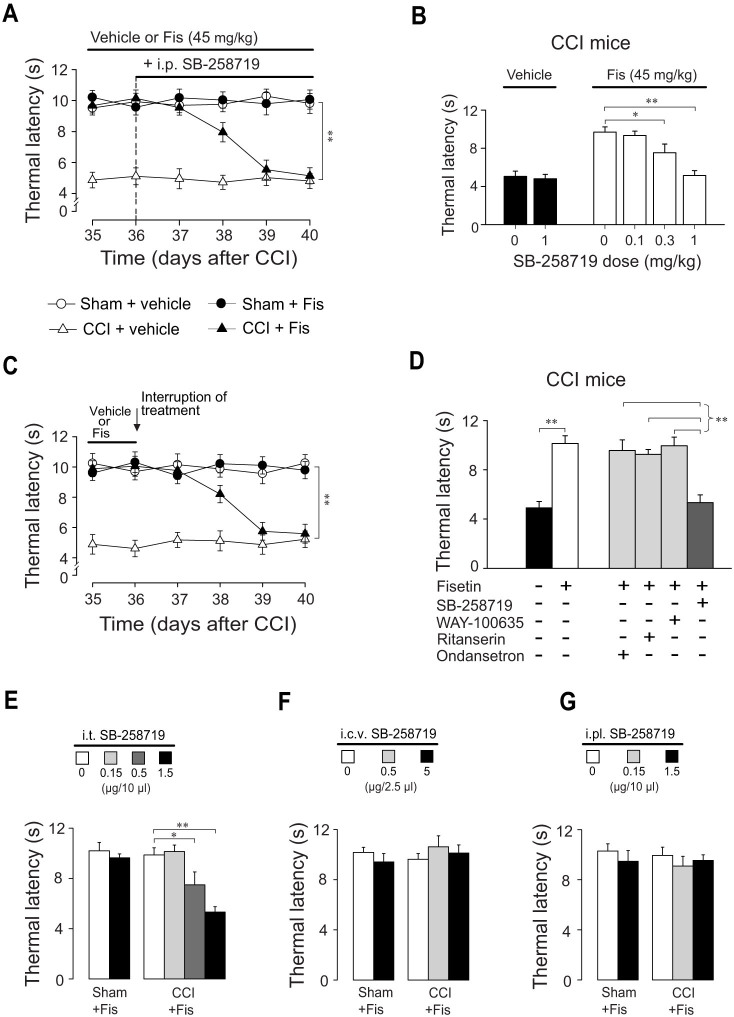
The antihyperalgesic effect of fisetin (Fis) in neuropathic mice is mediated by spinal 5-HT_7_ receptors. (A) Co-administration of 5-HT_7_ receptor antagonist SB-258719 (1 mg/kg, i.p.) abrogated fisetin antihyperalgesia in CCI mice, without influencing the thermal sensitivity in sham or vehicle-treated CCI mice. (B) SB-258719 (0.1, 0.3 and 1 mg/kg) dose-dependently counteracted the increase of thermal latency in Fis-treated CCI mice. (C) Effect of fisetin (45 mg/kg, p.o.) cessation on its therapeutic action against thermal hyperalgesia in CCI mice. Vehicle or fisetin administration was stopped following 21 days of treatment (i.e. day 36). (D) The comparison of the effect of co-administration of WAY-100635, ritanserin, ondansetron or SB-258719 on fisetin antihyperalgesia in CCI mice. (E) Intrathecal (i.t., 0.15, 0.5 and 1.5 μg in 10 μl) injection of SB-258719 dose-dependently decreased the thermal latency in Fis-treated CCI mice. (F) Effect of intracerebroventricular (i.c.v., 0.5 and 5 μg in 2.5 μl) injection of SB-258719 on the thermal latency in sham and CCI mice. (G) Effect of intraplantar (i.pl., 0.15 and 1.5 μg in 10 μl) injection of SB-258719 on the thermal latency in sham and CCI mice. (A) and (C) Open circles represent sham + vehicle group; solid circles represent sham + Fis group; open triangles represent CCI + vehicle group; solid triangles represent CCI + Fis group. Data are expressed as mean ± SEM (n = 9–12 per group), assessed by multifactor ANOVA followed by Duncan test.

To identify which subtypes of 5-HT receptors are involved in the antihyperalgesic effect of fisetin, we evaluated the effects of several selective 5-HT receptor antagonists on the antihyperalgesia by fisetin. These antagonists were: 5-HT_1A_ receptor antagonist WAY-100635 (1 mg/kg, i.p.), 5-HT_2A/C_ receptor antagonist ritanserin (4 mg/kg, i.p.), 5-HT_3_ receptor antagonist ondansetron (0.5 mg/kg, i.p.) and 5-HT_7_ receptor antagonist SB-258719 (1 mg/kg, i.p.). In CCI mice, the antihyperalgesic action of fisetin was abolished by repeated co-administration of the selective 5-HT_7_ receptor antagonist SB-258719 (F_4,188_ = 14.7, *p* < 0.01, Fig. 5A), even though the first co-administration had no acute suppressing effect (data not shown). After 4 days co-treatment, SB-258719 reversed the dose-dependent increase of thermal latency in CCI mice treated with fisetin (F_3,78_ = 42.4, *p* < 0.01; Fig. 5B), without influencing the thermal sensitivity of sham and vehicle-treated CCI mice (Fig. 5A and B). By contrast, the other 5-HT antagonists (WAY-100635, ritanserin and ondansetron) did not modify the antihyperalgesic effect of fisetin (Fig. 5D), further supporting the exclusive role for 5-HT_7_ receptors in mediating this action. In another set of mice repeatedly treated with fisetin, we interrupted the treatment in order to evaluate the delay before the spontaneous relapse of neuropathic hyperalgesia. This delay was analogous, in time course and modality, to that observed following systemic co-administration of SB-258719 (Fig. 5C).

To further investigate 5-HT_7_ receptors implication and locate their functional involvement in the antihyperalgesic effect of fisetin, we injected SB-258719 via three different routes of drug delivery (i.t., i.c.v. and i.pl.) and tested its effect on the antihyperalgesic effect of fisetin. As shown in Fig. 5E, i.t. injection of SB-258719 (1.5 μg in 10 μl) abrogated the therapeutic effect of fisetin on thermal hyperalgesia (F_2,116_ = 32.6, *p* < 0.01) in CCI mice, without influencing the measures in sham mice. Whereas, i.c.v. (5 μg in 2.5 μl) or i.pl. (1.5 μg in 10 μl) injection of SB-258719 did not modify the nociceptive responses to thermal stimuli in both sham and neuropathic mice.

### Chronic fisetin treatment ameliorated the co-morbidly behavioral symptoms of depression and anxiety in neuropathic mice

**Figure 6:**
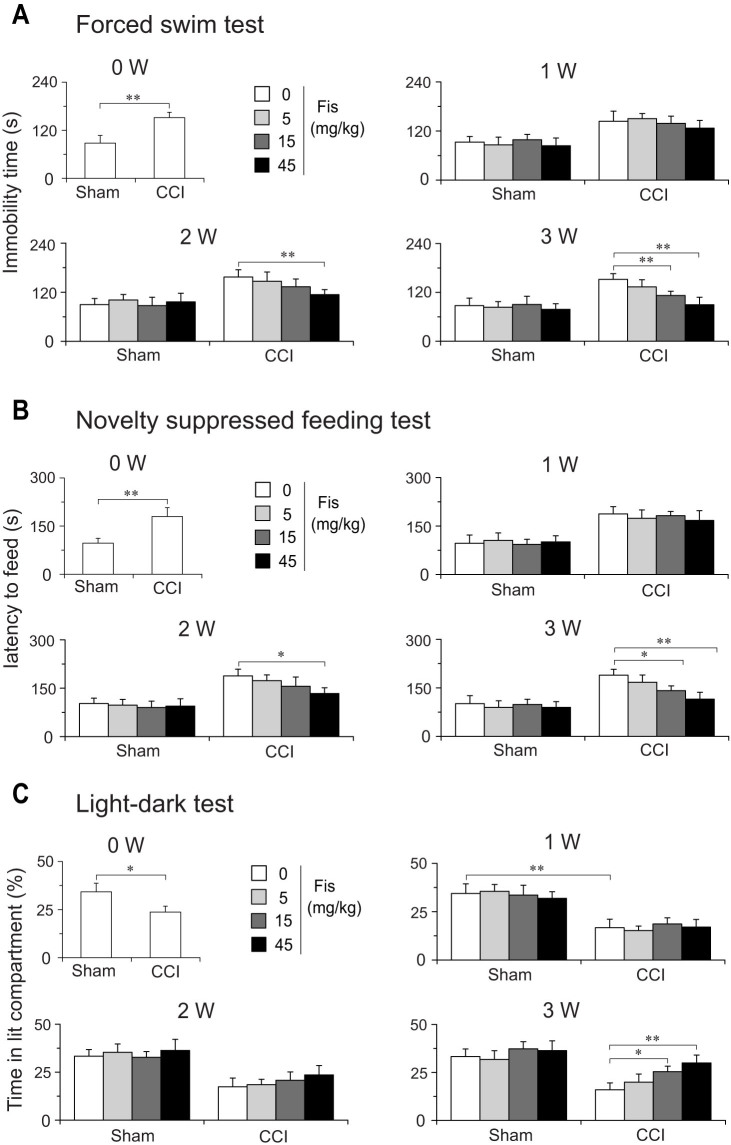
Chronic fisetin (Fis) treatment ameliorated the co-morbidly behavioral symptoms of depression and anxiety in mice with mononeuropathy. (A) In forced swim test (FST), chronic treatment with fisetin (5, 15 and 45 mg/kg, p.o., twice per day for 3 weeks) dose- and time-dependently decreased the immobility time in CCI mice, without influencing the measures in sham mice. (B) In novelty suppressed feeding test (NSFT), chronic treatment with fisetin (5, 15 and 45 mg/kg, p.o., twice per day for 3 weeks) dose- and time-dependently decreased the latency to feed in CCI mice, but not in sham mice. (C) Chronic treatment of CCI mice with fisetin (5, 15 and 45 mg/kg, p.o., twice per day for 3 weeks) dose- and time-dependently attenuated the anxiety-like behavior (decreased time in lit compartment) in light-dark test, but the same fisetin regimen did not alter the measures in sham mice. Data are expressed as mean ± SEM (n = 9–12 per group), assessed by multifactor ANOVA followed by Duncan test.

It has been reported that rodents (mice and rat) rendered neuropathic by peripheral injury manifested co-morbid depressive and anxiety-like behaviors^14^,^15^,^29^. After investigating the antinociceptive efficacy of fisetin in neuropathic mice, we hence examined its ability to affect the co-morbid depressive and anxiety-like behaviors induced by neuropathic pain. In our preliminary studies, we observed that the neuropathic mice developed co-morbid depressive and anxiety-like behaviors approximate 2–3 weeks following CCI surgery, as shown by prolonged immobility time in FST (a profile of depression-like behavior), increased latency to feed in NSFT (a profile indicative of both depression and anxiety-like behavior) and decreased lit compartment time in LDT (a profile of anxiety-like behavior). Although pain-related behaviors preceded the co-morbid depression and anxiety, the behavioral deficits in depression and anxiety tests were apparently noticed in neuropathic mice from 2 week to 6 week after CCI surgery (Fig. 6), suggestive of a triad of pain-depression-anxiety.

After 3 weeks treatment, fisetin dose-dependently attenuated in CCI mice the co-morbidly behavioral phenotypes of depression and anxiety. (a) In FST, fisetin (5, 15 and 45 mg/kg) dose-dependently decreased (by 13.7%, 26.1% and 41.8%, respectively) the prolonged immobility time (F_3,38_ = 12.1, *p* < 0.01; Fig. 6A); (b) In NSFT, fisetin (5, 15 and 45 mg/kg) dose-dependently reduced (by 10.9%, 24.3% and 37.9%, respectively) the latency to feed (F_3,39_ = 16.6, *p* < 0.01; Fig. 6B); (c) In LDT, fisetin (5, 15 and 45 mg/kg) dose-dependently increased (by 26.1%, 62.5% and 91.6%, respectively) the time stayed in lit compartment (F_3,40_ = 9.2, *p* < 0.01; Fig. 6C). These results clearly demonstrate that fisetin displays beneficial actions upon the co-morbid depression and anxiety-like behaviors specifically in mice with mononeuropathy. However, chronic treatment with fisetin did not affect the measures of depression and anxiety-like behaviors in sham mice.

**Figure 7:**
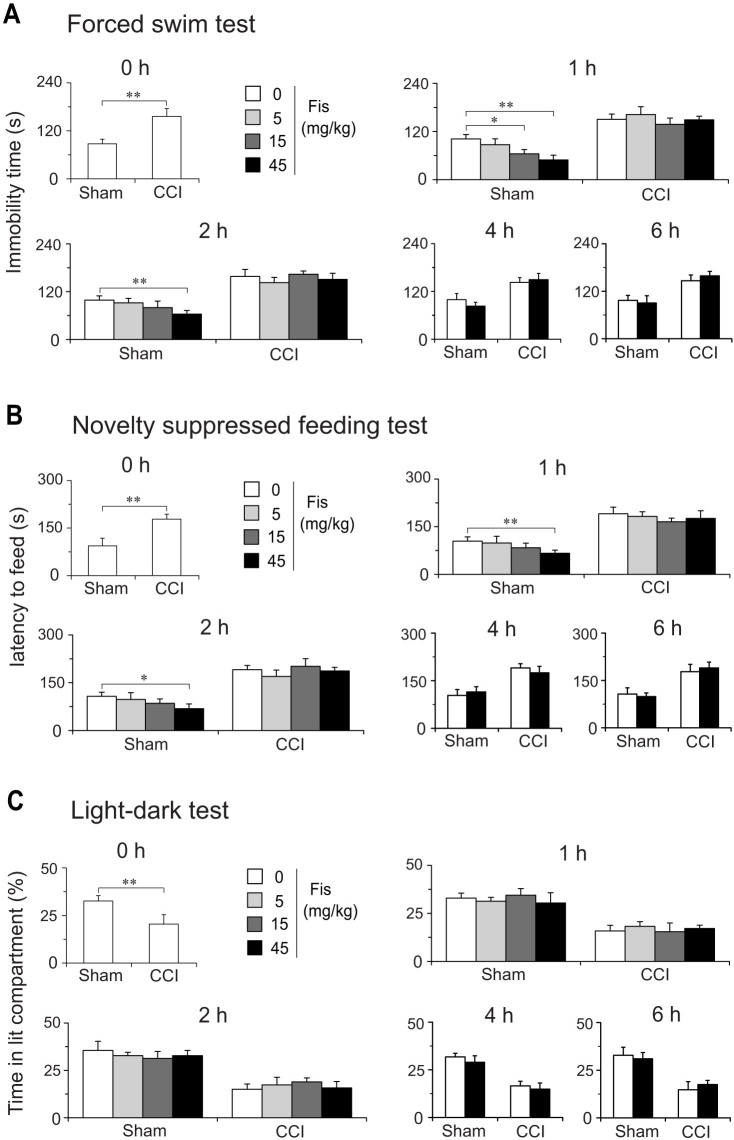
Effect of acute fisetin (Fis) administration on depression-like and anxiety-like behaviors in sham and CCI mice. Acute fisetin administration started 15 days after CCI surgery. Mice (sham or CCI) were administered with vehicle or fisetin (5, 15 and 45 mg/kg) and challenged in forced swim test (FST, A), novelty suppressed feeding test (NSFT, B) or light-dark test (LDT, C) at different time points (0, 1, 2, 4 and 6 h) after fisetin administration. (A) and (B) Acute fisetin administration (5, 15 and 45 mg/kg) displayed rapid onset antidepressant-like effect in sham rather than CCI mice, evidenced by reduced immobility time in FST (A) and a decrease of latency to feed in NSFT (B). This action is dose dependent and seems to be transitory (lasts for no more than 4 h). (C) In light-dark test, acute fisetin administration (5, 15 and 45 mg/kg) did not show any anxiolytic activity in both sham and CCI mice. Data are expressed as mean ± SEM (n = 9–12 per group), assessed by multifactor ANOVA followed by Duncan test.

We also evaluated the effects of acute fiseitn treatment on the depression-like and anxiety-like behaviors in sham and CCI mice. In depression-related behavioral tests (FST and NSFT), acute fisetin treatment exhibited positive activities in sham-operated mice (Fig. 7A and B). But this action is a short-term transitory one (persisted no more than 4 h) and thus cannot be considered as a real “therapeutic action”. Unexpectedly, the same acute fisetin regimen (at the same dose range of 5–45 mg/kg) did not show any anti-depression efficacy in CCI mice, implying its acute anti-depression potential is blunted in a neuropathic condition. In LDT, acute fisetin treatment did not show anti-anxiety like effect in both sham and CCI mice (Fig. 7C).

### Effects of chronic fisetin treatment on locomotor activity in sham-operated and CCI-injured mice

**Figure 8:**
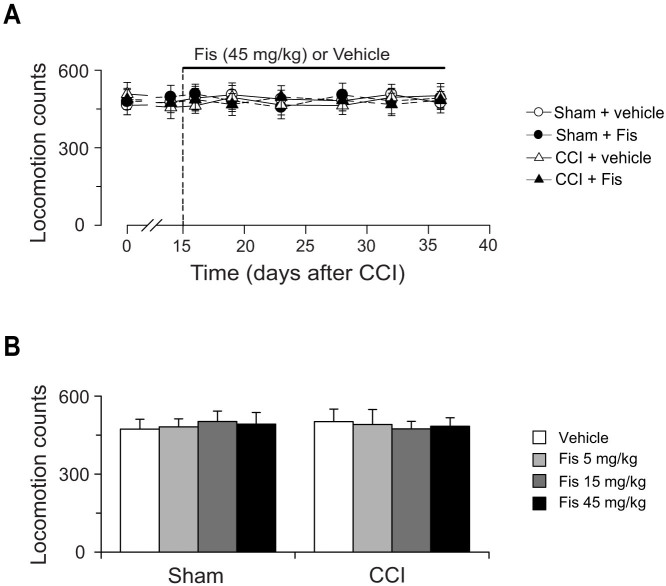
Effect of chronic fisetin (Fis) treatment on locomotor activity in sham and CCI mice. (A) Time-course of Fis (45 mg/kg) action on locomotor activity in sham and CCI mice. Open circles represent sham + vehicle group; solid circles represent sham + Fis group; open triangles represent CCI + vehicle group; solid triangles represent CCI + Fis group. (B) Fis within the dose range studied (5–45 mg/kg) did not impact on the locomotor activity in both sham and CCI mice. Data are expressed as mean ± SEM (n = 9–12 per group), assessed by multifactor ANOVA followed by Duncan test.

As shown in Fig. 8, peripheral nerve injury (CCI) did not alter locomotor activity in mice. In both sham and CCI mice, chronic fisetin treatment did not influence the measures in locomotor activity test. The paucity of locomotor inhibition by fisetin indicates its antidepressant-like effect, expressed by attenuation of protracted immobility time (FST), should not be explained by sedative or motor abnormality that cause false positive results in FST.

## Discussion

In the present study, we show that fisetin, when chronically and orally administered in mice with mononeuropathy, exerted antihyperalgesic effects. This action is modality and time dependent since it is sensitive to thermal (heat) stimuli but not to mechanical one, and is present following chronic rather than acute fisetin treatment. Mechanistically, the antihyperalgesia by fisetin may depend on the activation of descending serotonergic system, which is coupled with spinal 5-HT_7_ receptors. In addition, persistent mononeuropathy elicited in mice co-morbidly behavioral symptoms of depression and anxiety and these phenotypes can also be palliated by chronic treatment with fisetin.

The CCI model from Bennett and Xie^16^ was selected in this study since it can produce in rodents persistent and stable pain-related behaviors and thus is widely used as a canonical animal model of neuropathic pain^32^. We used Hargreaves test and von Frey test to assay pain-related behaviors in mice because the two procedures can examine mouse behaviors recapitulating clinical signs and symptoms in patients with neuropathic pain, i.e. enhanced response to noxious stimuli (hyperalgesia) and a pain response to previously innocuous stimuli (allodynia). Likewise, a triad behavioral tests consisting of forced swim test (FST), novelty suppressed feeding test (NSFT) and light-dark test (LDT) were employed to characterize the co-morbid symptoms of depression and anxiety in neuropathic mice, and these procedures are pharmacologically validated and relatively easy (not heavy procedures to set up and operate) to examine depression-like and anxiety-like behaviors in rodents and screen antidepressants and anxiolytics in preclinical studies. Here, we show that the mice with mononeuropathy exhibited synchronous depressive-like and anxiety-like behaviors, which is consistent with other reports^14^,^15^,^29^ and indicates that the mouse neuropathic model produced by peripheral nerve injury (CCI model) is suitable for mimicking the co-morbid mood disorders provoked by neuropathic pain.

The main finding of the present study is the ability of fisetin to induce relief of thermal (heat) hyperalgesia in neuropathic mice following 3 weeks of oral treatment. To our best knowledge, it is the fist report to demonstrate the antinociceptive activity of fisetin and its antinociceptive action incorporates several characteristic aspects. Firstly, the antihyperalgesia of fisetin is a reversing action (not a prophylactic one) as the treatment started on the 15^th^ day following CCI-lesion when the pain-related behaviors had been established, thus implying its potential utility in the development of novel and clinically relevant drugs for the treatment of neuropathic pain. Accordingly, it would be intriguing to know whether fisetin can prevent the development of chronic pain by preemptive administration. Secondly, its antinociceptive action is modality and time dependent, inasmuch as it works in fighting thermal hyperalgesia but is actually not responsive to mechanical hypersensitivity, and this antihyperalgesia was observed following repeated but not single dose fisetin regimen. Thirdly, it is notable to point out that the antihyperalgesia by fisetin shown in the present study should be a genuine one, since the nociceptive tests were performed on the next morning following the last fisetin administration and thus the antihyperalgesic action is present for at least one day. This point is important and indicates that the antihyperalgesia provided by fiseitn is persistent but not transient. Lastly, our results delineates the spinal site of fisetin antinociception, which is similar to previous reports concerning action site of some antidepressant drugs^20^,^21^ and thus provides new insights into the mechanisms underlying fisetin antinociception.

The descending monoamine pathway, especially that involving noradrenergic and serotonergic transmission, is a major component of the endogenous pain modulatory system^12^. Our previous study revealed that fisetin escalated aminergic tone in mice, which correlates with its anti-depression effects^11^. We thus speculated fisetin treatment can activate the descending monoamine system and this monoaminergic mechanism may account for its currently antihyperalgesic efficacy. This assumption is confirmed by the present study, as fisetin treatment markedly increased the spinal monoamine (serotonin) levels and the ratio of 5-HT/5-HTP, and concomitantly depressed spinal MAO-A. But a mechanistic concern is raised as whether the monoamine-related changes are causal to the therapeutic action of fisetin or these outcomes are only a consequence of pain relief. To address this question, we chemically ablated spinal 5-HT or NA in mice and evaluated whether these procedures can impact on the antihyperalgesic action of fisetin. Our results clearly show chemical depletion of spinal 5-HT but not NA abrogated the antihyperalgesic action of fisetin and thus implicate an exceptional serotonergic mechanism underlying fisetin antihyperalgesia. This assertion is strengthened by another fact that pretreatment with 5-HTP, a precursor of 5-HT, potentiated fisetin antihyperalgesia.

To further investigate the pharmacological mechanisms behind fisetin action, we tested different 5-HT receptor antagonists to identify which subtypes of 5-HT receptors are responsible for the antihyperalgesic effects of fisetin. Our results unequivocally show co-administration of the 5-HT_7_ receptor antagonist SB-258719 completely abolished the antihyperalgesic action of fisetin, but it is not the case for other 5-HT receptor antagonists (WAY-100635, ritanserin and ondansetron). These observations strongly argue for the key role of 5-HT_7_ receptors underlying fisetin antihyperalgesia and are congruent with recent studies reporting the engagement of 5-HT_7_ mechanism in the initiation and maintenance of neuropathic pain^19^,^33^. More precisely, we localized the responding 5-HT_7_ receptors to spinal but not supraspinal or peripheral level, as i.t., but not i.c.v. or i.pl., injection of SB-258719 counteracted the antihyperalgesic action of fisetin. This is supported by immunocytochemical studies showing that 5-HT_7_ receptors are localized in the superficial layers of the spinal dorsal horn, a key region for pain modulation^19^. Therefore, it is feasible to attribute the antihyperalgesic mechanism of fisetin to the enhanced descending 5-HT tone, which was coupled with activation of spinal 5-HT_7_ receptors. Of note, the inhibition of antihyperalgesic action of fisetin by blockade of 5-HT_7_ receptors requires a repeated co-administration process, which is reminiscent, as for modality and time-course, of the relapse of hyperalgesia after fisetin withdrawal. This temporal delay suggests that the escalation of serotonergic tone may be a primary or upstream target for fisetin action.

Recently, we also screened the antinociceptive bioactivities of several other naturally occurring compounds such as curcumin^19^ and resveratrol^34^ in the mouse neuropathic pain model (CCI model). Like fisetin, curcumin and resveratrol also possess antinociceptive activities when orally and chronically dosed in neuropathic mice. Although the three compounds share monoaminergic mechanism underlying their antinociceptive actions, the receptor selectivity for these molecules is not always consistent (with curcumin influencing 5-HT_1_, while fisetin and resveratrol coupled with 5-HT_7_). This is interesting and worthy of further investigations. In light of the present results and those obtained in previous reports^19^,^34^, some points and implications should be taken into account in future studies. For example, is there a link between MAO-A inhibition and the predominant 5-HT_7_ selectivity of fisetin and resveratrol? Are other mechanisms such as inhibition of 5-HT re-uptake and increase of 5-HT release involved in the antinociceptive actions exerted by these compounds?

Patients with chronic neuropathic pain often suffer from affective disorders like depression and anxiety^13^, but relative few studies evaluated the effects of drugs on these co-morbidly emotional disabilities. Here, we also behaviorally characterized the co-morbid symptoms of depression and anxiety in neuropathic mice and investigated the actions of fisetin. Consistent with other preclinical studies^14^,^15^,^29^, we also observed co-morbidly behavioral phenotypes of depression and anxiety in neuropathic mice. However, the depression and anxiety-like behaviors in CCI mice developed relatively chronic (about 2–3 weeks) compared with their rapid-onset nociceptive behaviors (no more that 5 days), suggestive of long-term events of neural plasticity being involved in the initiation of these co-morbid mood disorders. Following chronic (3 weeks) but not acute treatment, fisetin remarkably ameliorated in a dose-dependent manner the behavioral phenotypes of depression and anxiety in neuropathic mice. This delayed efficacy of fisetin echoes putatively altered neural plasticity caused by chronic pain and is consistent with the clinical setting of repeated antidepressant drugs treatment^35^. Of note, the anti-depression and anti-anxiety following chronic fisetin administration are not generalized to sham-operated mice, implying these actions are associated, to some extend, with its concurrently antinociceptive effect. In contrast, the single dose fisetin regimen produced obvious antidepressant-like (but not anxiolytic) effect in sham-operated mice, which is generally congruent with our previous observations on the anti-depression activity of fisetin^11^, but this action is transient (persisted no more than 4 h) and actually lost in CCI mice. Thus, it could be inferred that chronic rather than acute fisetin regimen may benefit symptoms of depression and anxiety caused by persistent neuropathic pain. Although further studies are needed to address the detailed mechanism for fisetin anti-depression and anti-anxiety in the context of mononeuropathy, the beneficial actions of fisetin on these co-morbid mood disorders may favor its possible utility in fighting chronic neuropathic pain.

In conclusion, the present study demonstrates that fisetin, when administered orally and chronically in neuropathic mice, can correct their thermal (heat) hyperalgesia, indicating a potential use of this naturally occurring molecule in the clinical setting of chronic neuropathic pain. Although further studies should be undertaken to fully elucidate the precise mechanisms for fisetin action (especially the predominant receptor selectivity of 5-HT_7_), we propose that the enhanced spinal 5-HT tone (coupled to 5-HT_7_ receptors) may exclusively account for the mechanism behind fisetin antihyperalgesia. Over time, the mice with mononeuropathy developed behavioral symptoms of depression and anxiety. Our findings also confirm that chronic fisetin treatment can significantly improve aspects of these co-morbidly behavioral endophenotypes.

## Author Contributions

X.Z. designed this study. X.Z., C.W., W.G.C., Q. M. and W.H.Z. performed the experiments. X.Z., C.W. and W.H.Z. analyzed the data. X.Z. wrote the paper. All authors read and approved the paper.

## Figures and Tables

**Table 1:** Effect of chronic fisetin treatment on monoamines (metabolites) in the spinal cord of Sham and CCI mice

	Concentration (ng/g)					
Group	5-HT	5-HIAA	5-HIAA/5-HT	NA	Dopamine	DOPAC
Sham mice						
Vehicle	734.8 ± 31.0	362.9 ± 25.4	0.49 ± 0.06	566.0 ± 21.5	73.1 ± 6.6	26.7 ± 4.3
Fis-45 mg/kg	765.4 ± 40.2	340.0 ± 21.0	0.44 ± 0.07	582.9 ± 28.1	77.5 ± 5.9	23.8 ± 3.8
CCI mice						
Vehicle	447.5 ± 38.6##	381.1 ± 24.8	0.85 ± 0.05##	531.4 ± 22.8	68.4 ± 5.5	28.1 ± 3.9
Fis-5 mg/kg	478.9 ± 34.1	393.7 ± 28.6	0.82 ± 0.07	549.9 ± 18.0	72.0 ± 6.7	27.2 ± 4.6
Fis-15 mg/kg	571.3 ± 45.6*	372.6 ± 19.9	0.65 ± 0.06**	573.4 ± 29.1	69.8 ± 4.3	23.8 ± 5.4
Fis-45 mg/kg	694.3 ± 39.8**	358.4 ± 26.0	0.51 ± 0.07**	590.7 ± 24.3*	74.3 ± 6.0	24.5 ± 4.7

Fis: fisetin. Values were expressed as mean ± SEM of 6–9 mice.

^##^*p* < 0.01, compared with vehicle treated sham mice.

*p < 0.05 and **p < 0.01, compared with vehicle treated CCI mice.

**Table 2:** Effect of chronic fisetin treatment on spinal monoamine oxidase activities in sham and CCI mice

	Monoamine oxidase-A activity (nmol/30 min/mg protein)		Monoamine oxidase-B activity (nmol/30 min/mg protein)	
Group	Sham mice	CCI mice	Sham mice	CCI mice
Vehicle	122.7 ± 5.8	157.2 ± 5.5##	143.5 ± 7.1	149.3 ± 6.2
Fis-5 mg/kg	-	149.2 ± 6.3	-	151.3 ± 7.8
Fis-15 mg/kg	-	136.0 ± 4.9**	-	143.9 ± 5.4
Fis-45 mg/kg	115.5 ± 7.2	125.3 ± 6.8**	138.0 ± 6.7	145.6 ± 6.6

Fis: fisetin. -: data not available. Values were expressed as mean values ± SEM of 6–9 mice.

^##^*p* < 0.01, compared with vehicle treated Sham mice.

**p < 0.01, compared with vehicle treated CCI mice.
